# Gastrointestinal herpes zoster in a patient on infliximab: a case report

**DOI:** 10.1093/omcr/omaf171

**Published:** 2025-09-28

**Authors:** Imane El Hamraoui, Houda El Hiouy, Salma Mechhor, Manal Cherkaoui El Malki, Hicham El Bacha, Nadia Benzzoubeir, Fouad Zouaidia, Ikram Errabih

**Affiliations:** Department of Gastroentero-Hepatology and Proctology “Medecine B”, Ibn Sina University Hospital, Mohammed V University, Abderrahim Bouabid avenue, Souissi, Rabat 10100, Morocco; Department of Gastroentero-Hepatology and Proctology “Medecine B”, Ibn Sina University Hospital, Mohammed V University, Abderrahim Bouabid avenue, Souissi, Rabat 10100, Morocco; Department of Gastroentero-Hepatology and Proctology “Medecine B”, Ibn Sina University Hospital, Mohammed V University, Abderrahim Bouabid avenue, Souissi, Rabat 10100, Morocco; Department of Gastroentero-Hepatology and Proctology “Medecine B”, Ibn Sina University Hospital, Mohammed V University, Abderrahim Bouabid avenue, Souissi, Rabat 10100, Morocco; Department of Gastroentero-Hepatology and Proctology “Medecine B”, Ibn Sina University Hospital, Mohammed V University, Abderrahim Bouabid avenue, Souissi, Rabat 10100, Morocco; Department of Gastroentero-Hepatology and Proctology “Medecine B”, Ibn Sina University Hospital, Mohammed V University, Abderrahim Bouabid avenue, Souissi, Rabat 10100, Morocco; Department Pathology, Ibn Sina University Hospital, Mohammed V University, Abderrahim Bouabid avenue, Souissi,Rabat 10100, Morocco; Department of Gastroentero-Hepatology and Proctology “Medecine B”, Ibn Sina University Hospital, Mohammed V University, Abderrahim Bouabid avenue, Souissi, Rabat 10100, Morocco

**Keywords:** VZV, infliximab, gastrointestinal involvement, acyclovir

## Abstract

Varicella-zoster virus (VZV) reactivation in immunocompromised patients may present atypically, including with severe gastrointestinal involvement. We present a case of a 36-year-old male with ileocolic Crohn’s disease, who, following Infliximab (IFX) therapy, developed severe gastrointestinal symptoms. Subsequent investigations confirmed a disseminated VZV infection with esophagogastric and duodenal involvement. Our case highlights the crucial need for heightened clinical awareness regarding unusual VZV presentations in immunocompromised patients, especially those receiving TNF- α inhibitors such as Infliximab, known to increase the risk of VZV reactivation. Given the potentially high mortality rate (approximately 40%) associated with gastrointestinal VZV involvement, prompt and aggressive antiviral treatment is imperative.

## Introduction

Varicella-zoster virus (VZV), a neurotropic virus, establishes latency in sensory, enteric, and other autonomic neurons following primary varicella infection. Reactivation of VZV can result in a distinct gastrointestinal presentation -"enteric zoster”- which may occur with or without cutaneous manifestations [1]. This form of reactivation caaries a significant risk of mortality, particularly in immunocompromised individuals [2]. We describe a rare case of disseminated VZV infection with esophagogastric and duodenal involvement in a patient with Crohn’s disease (CD) receiving infliximab (IFX), managed at the Gastroentero-Hepatology and Proctology Department of Ibn Sina Hospital in Rabat, Morocco.

## Case presentation

A 36-year-old patient, with a past history of digestive tuberculosis diagnosed in 2020, was newly diagnosed in 2023 with stenosing and fistulizing ileocolic Crohn’s disease. He underwent an extended right ileocolectomy up to the transverse colon, followed by a mechanical ileo-transverse anastomosis and surgical disconnection of the fistulous tracts. Postoperatively, the patient developed enterocutaneous fistulas.

Combined immunosuppressive therapy with Azathioprine and IFX was initiated. Pre-immunosuppression screening showed negative results for hepatitis B, hepatitis C, human immunodeficiency virus (HIV), and syphilis. IgM antibodies for herpes viruses including VZV, herpes simplex virus (HSV), cytomegalovirus (CMV), and Epstein–Barr virus (EBV) were negative, while IgG antibodies were all positive, indicating past exposure.

Urine cytobacteriology, stool parasitology and fecal culture were all negative.

An Interferon-Gamma Release Assay was negative as well.

Initial laboratory tests showed a hemoglobin level of 11 g/dl, a leukocyte count of 7400/μl, and a platelet count of 232 000/μl. Liver enzymes including AST, ALT, GGT, and alkaline phosphatase, were within normal ranges. C-reactive protein (CRP) was measured at 5 mg/l.

Azathioprine was therefore initiated at a dose of 2.5 mg/kg/day but was discontinued after two weeks due to drug-induced toxidermia, presenting as a maculopapular rash associated with chills and signs of systemic inflammation.

**Figure 1:**
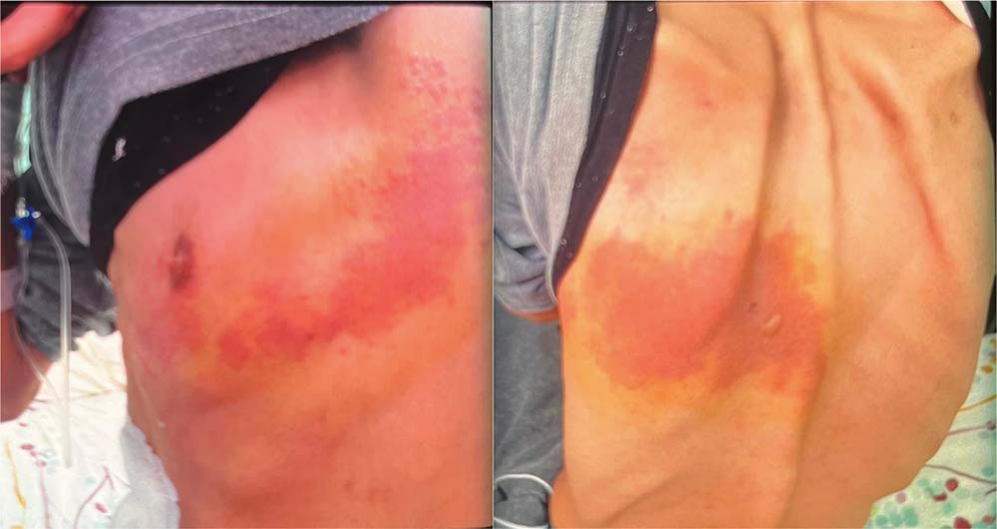
Herpes zoster hyperemic lesions in the left intercostal zone with one vesicle appearing on the dorsal surface.

After full recovery from the toxidermic reaction, the first infusion of IFX was administered at a dose of 5 mg/kg without immediate complications. Two days later, the patient presented with significant postprandial vomiting and severe epigastric pain. On the following day, a painful, erythematous, and hyperemic rash appeared on the left intercostal area, consistent with a dermatomal distribution, followed by the emergence of a few vesicular lesions on the dorsal surface (Fig. 1). He also had a low-grade fever at 38.2° and chills. The clinical presentation was suggestive of intercostal herpes zoster.

**Figure 2:**
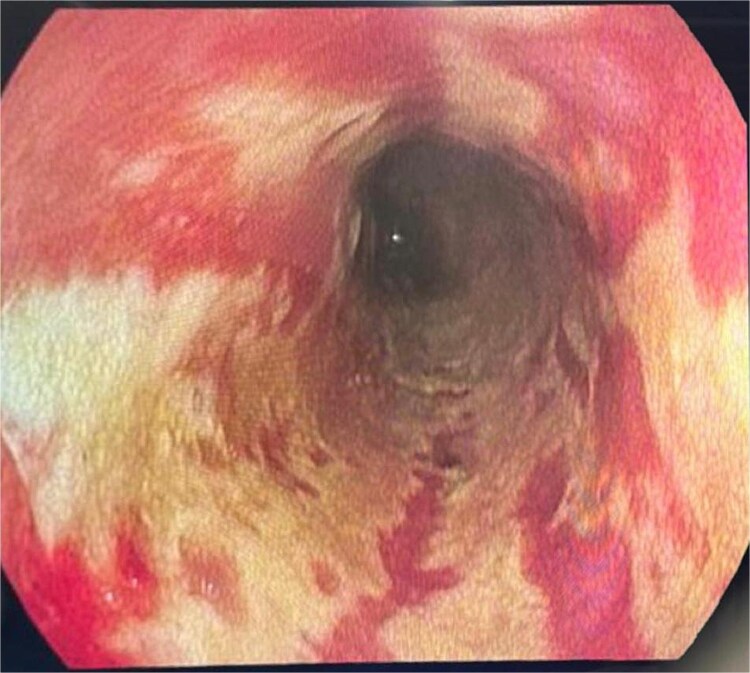
Stade D esophagitis with circumferential ulcerations along the esophagus.

**Figure 3:**
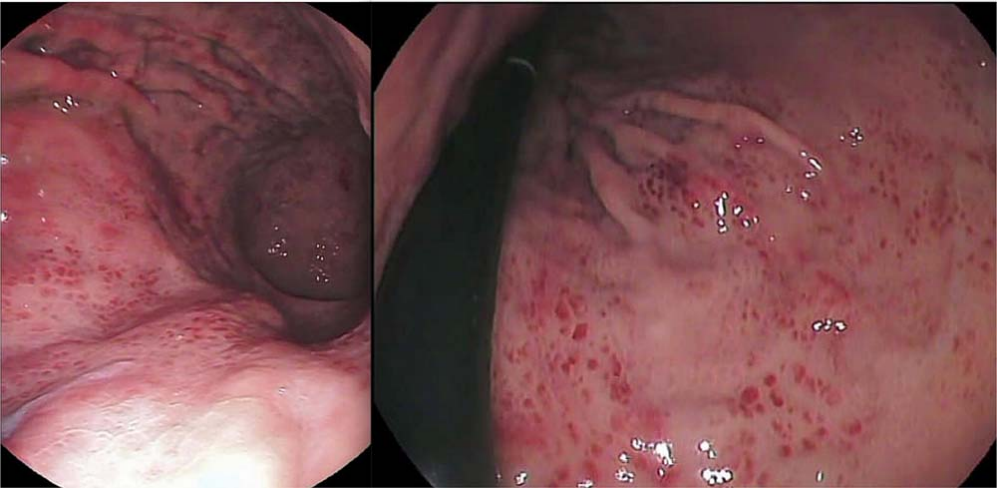
Erythematous petechial pangastritis.

Laboratory tests showed evidence of systemic inflammation: leukocytosis at 29000/ml with neutrophil predominance, thrombocytosis at 631000/ml, and an elevated CRP level of 120 mg/l. VZV PCR testing was positive in both blood and vesicular fluid. Due to persistent digestive symptoms, an esophagogastroduodenoscopy (EGD) was performed revealing a stage D esophagitis with linear longitudinal circumferential ulcerations along the esophagus (Fig. 2), an erythematous-petechial pangastritis (Fig. 3) and an erythematous bulbitis and duodenitis. There was no significant fluid retention observed during endoscopy.

**Figure 4:**
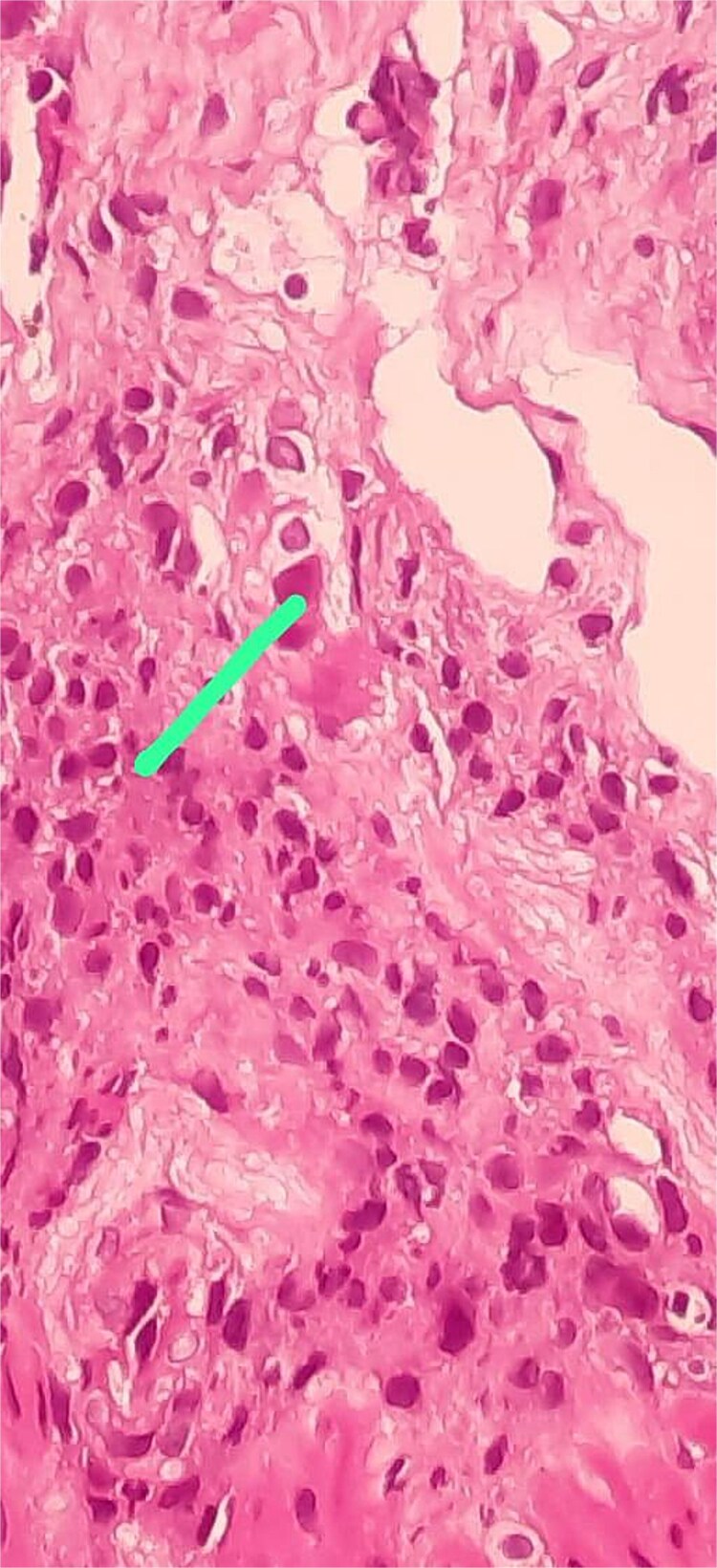
(Gx40 HE) viral inclusion within an endothelial cell.

Histopathological findings revealed severe active gastritis involving the antrum and fundus, with negative *Helicobacter pylori* testing. Herpetiform viral inclusions were identified in the antral mucosa, consistent with VZV-associated digestive lesions (Fig. 4).

The patient was initially treated with intravenous Acyclovir 10 mg/kg every 8 hours and transferred to the infectious diseases department for further management.

## Discussion

VZV reactivation typically manifests as herpes zoster, confined to the skin and presenting as a metameric vesicular rash [3]. However, in 3 to 15% of cases, VZV reactivation takes the form of a disseminated infection with visceral involvement, affecting lungs, central nervous system, liver, and more rarely the pancreas or digestive tract [4].

The frequency of specific digestive tract involvement during VZV reactivation is unknown. A literature review found fewer than 30 reported cases (cases where diagnosis was confirmed by endoscopy or autopsy). The majority of these patients had hematological diseases treated with chemotherapy and stem cell allografts or autografts. Symptoms commonly described include fever and epigastric abdominal pain, associated with nausea or vomiting. Endoscopic findings typically reveal digestive mucosal ulcerations primarily in the stomach, but also in the esophagus, duodenum, small intestine, or colon [2]. We report here the first documented case occurring in the context of Crohn’s disease under Infliximab therapy (Table 1).

**Table 1:** Observations of digestive mucosal involvement during disseminated VZV infection (digestive involvement proven by endoscopy or autopsy) [2].

Reference	N	Age	Associated pathology	Digestive localization	Outcome
David and al, 1998	10	40 [27–56]	Bone marrow transplant (two autografts + eight allografts)	Stomach	5 favorable
Yagi et al., 2000	2	30, 32	Chronic myeloid leukemia and allograft, acute lymphoblastic leukemia and allograft	Stomach, esophagus, small intestine	1 favorable, 1 death
Itoh and al., 2001	2	17,40	Medullary aplasia and allograft, acute leukemia and allograft	Stomach	Favorable
Khilnani and Keller, 1971	1	61	Polycythemia vera	Stomach	Death
Sherman and al., 1991	1	38	Asthma, corticosteroid therapy	Small intestine	Death
McCluggage and al., 1994	1	33	Hodgkin’'s disease, autograft	Stomach	Favorable
Stratman, 2002	1	77	Non-Hodgkin lymphoma	Stomach	Favorable
Scholl and al., 2006	1	54	Non-Hodgkin lymphoma	Stomach	Favorable
Rivera-Vaquerizo and al., 2001	1	41	Acute leukemia, allograft	Stomach	Favorable
Yamazaki and al., 2004	1	34	Acute leukemia, allograft	Esophagus, stomach, small intestine	Death
Leena and al., 2006	1	53	Myeloma, autograft	Stomach, duodenum, esophagus	Favorable
Nomdedéu and al., 1995	1	ND	Hodgkin’'s disease, autograft	Colon	Death
Sanz Moreno and al., 1996	1	66	Multiple myeloma	Small intestine, duodenum	Death
Precupanu and al., 2009	1	52	Non-Hodgkin lymphoma, autograft	Colon	Favorable
Milligan and al., 2012	1	18	Common variable immunodeficiency	Stomach	Favorable
Takatoku and al., 2004	1	54	Acute leukemia, allograft	Stomach	Favorable
Scholl and al., 2006	1	45	Non-Hodgkin lymphoma, autograft	Stomach	Favorable
Serris and al, 2014	1	76	Chronic lymphocytic leukemia	Stomach	Favorable
Reported case	1	36	Crohn disease under INFLIXIMAB treatment	Stomach, duodenum, esophagus	Favorable
Total	30	Median 44	Hemopathy 27/30 (90%)	Esophagus 5/30 (16,7%)	Deaths 11/30
		[17–77]	Crohn disease under INFLIXIMAB treatment 1/30 (3,3 %)	Stomach 26/30 (86,7%)	
			Other pathology 2/30 (6,7 %)	Duodenum 3/30 (10%)	
				Small intestine 5/30 (16,7%)	
				Colon 2/30 (6,7%)	

Most patients first develop digestive symptoms, followed by fever and the characteristic vesicular rash within an average of six days. The vesicular rash may be absent during disseminated VZV reactivation, and this deceptive form can delay diagnosis, thus worsening the prognosis [3, 5].

VZV reactivation forms with digestive involvement are particularly severe, with a mortality rate around 40%. Due to this severity, some authors recommend the empirical initiation of antiviral treatment with acyclovir as soon as disseminated VZV infection is suspected [3, 5, 6].

The risk of VZV reactivation is described in patients undergoing TNF- α therapy. It varies among different TNF- α agents. A significant association has been observed between herpes zoster and monoclonal TNF- α antibodies, particularly with IFX, but not with etanercept (ETN). It appears that the risk of VZV reactivation is higher with monoclonal antibodies. Among patients receiving TNF- α, monoclonal antibodies, especially IFX, have been significantly associated with an increased risk of herpes zoster [7].

Patients with autoimmune disease are at increased risk of VZV from both the underlying disease process itself and the use of immunomodulation. According to the guidelines from the CDC and ECCO, the live-attenuated VZV vaccine (ZVL) is contraindicated in immunocompromised patients, including those receiving biologic therapies or immunosuppressive treatments, due to the heightened risk of complications and reactivation of the virus [8, 9].

The recombinant zoster vaccine (RZV) is well tolerated in patients receiving immunosuppressive therapies or biologic treatments (such as IFX). It can be administered to patients at least 2 weeks prior to starting IFX, including those with positive IgG for VZV [10].

ACIP (The Advisory Committee on Immunization Practices) recommends 2 doses of RZV for the prevention of shingles and related complications in adults aged ≥19 years who are or will be immunodeficient or immunosuppressed because of disease or therapy. When possible, RZV should be administrated prior to initiation of IFX. If not possible, it can be administrated when IFX is anticipated to be low [11].

However, Jeffrey R. Curtis and al suggested in their study that ZVL was safe and had reasonable short-term effectiveness in participants receiving TNF- α. In addition, a significant increase in the immune response was observed. Moreover, the RZV is not available in many countries; in those areas, ZVL will continue to remain relevant from a public health perspective. Therefore, the use of this ZVL in TNF- α treated patients may be a reasonable option, especially in the absence of an alternative zoster vaccine [12].

In Morocco, neither the live-attenuated zoster vaccine (ZVL) nor the recombinant zoster vaccine (RZV) are currently available. Thus, no zoster vaccination options are accessible to immunocompromised patients at this time.

Antiviral prophylaxis, generally with low-dose valacyclovir, may be considered in select patients under TNF- α who are not candidates for RZV or who have had recurrences despite full immunization [13].
